# The superoxide dismutase mimetic tempol does not alleviate glucocorticoid‐mediated rarefaction of rat skeletal muscle capillaries

**DOI:** 10.14814/phy2.13243

**Published:** 2017-05-22

**Authors:** Erin R. Mandel, Emily C. Dunford, Ghoncheh Abdifarkosh, Patrick C. Turnbull, Christopher G. R. Perry, Michael C. Riddell, Tara L. Haas

**Affiliations:** ^1^ School of Kinesiology and Health Science and the Muscle Health Research Centre York University Toronto Ontario Canada

**Keywords:** Corticosterone, endothelium, reactive oxygen species, skeletal muscle blood flow

## Abstract

Sustained elevations in circulating glucocorticoids elicit reductions in skeletal muscle microvascular content, but little is known of the underlying mechanisms. We hypothesized that glucocorticoid‐induced oxidative stress contributes to this phenomenon. In rats that were implanted with corticosterone (CORT) or control pellets, CORT caused a significant decrease in muscle glutathione levels and a corresponding increase in protein carbonylation, an irreversible oxidative modification of proteins. Decreased endothelial nitric oxide synthase and increased endothelin‐1 mRNA levels were detected after 9 days of CORT, and blood flow to glycolytic muscles was diminished. Control and CORT rats were treated concurrently with drinking water containing the superoxide dismutase mimetic tempol (172 mg/L) or the *α*‐1 adrenergic receptor antagonist prazosin (50 mg/L) for 6 or 16 days. Both tempol and prazosin alleviated skeletal muscle protein carbonylation. Tempol failed to prevent CORT‐mediated capillary rarefaction and was ineffective in restoring skeletal muscle blood flow. In contrast, prazosin blocked capillary rarefaction and restored skeletal muscle blood flow to control levels. The failure of tempol to prevent CORT‐induced skeletal muscle microvascular rarefaction does not support a dominant role of superoxide‐induced oxidative stress in this process. Although a decrease in protein carbonylation was observed with prazosin treatment, our data suggest that the maintenance of skeletal muscle microvascular content is related more closely with counteracting the CORT‐mediated influence on skeletal muscle vascular tone.

## Introduction

Glucocorticoids (GC) are steroid hormones that have been shown to induce the loss of preexisting skeletal muscle capillaries (Shikatani et al. 2012; Mandel et al. 2016). This decline in skeletal muscle microvascular content, referred to as capillary rarefaction, exerts deleterious influences locally by perturbing the capacity to maintain appropriate exchange of nutrients and waste products, which can impair whole body metabolism. The resultant increase in vascular resistance to blood flow also has been predicted to lower tissue perfusion and increase the risk of ischemic damage (Hudetz 1993). Furthermore, the loss of skeletal muscle microvessels is believed to increase total peripheral vascular resistance (Greene et al. 1989; Humar et al. 2009; Heuslein et al. 2015), which could promote the development of systemic hypertension.

Capillary rarefaction is thought to be the consequence of an enhanced rate of endothelial cell death in combination with insufficient compensatory cell proliferation and angiogenesis (Gobé et al. 1997; Goligorsky 2010), although the causal mechanisms behind GC‐induced capillary rarefaction remain poorly defined. An interesting observation is that GC administration is associated with an increased production of reactive oxygen species, particularly superoxide radicals (Iuchi et al. 2003). Oxidative stress resulting from excessive levels of ROS has been causally implicated in a variety of related vascular pathologies, including endothelial cell dysfunction, excessive vasoconstriction, inward arterial remodeling, capillary rarefaction and the development of hypertension (Kobayashi et al. 2005; Montezano and Touyz 2014; Staiculescu et al. 2014). A major effect of excess ROS is the restraint of local tissue blood flow. For example, superoxide radicals promote vasoconstriction through decreasing nitric oxide (NO) bioavailability (Iuchi et al. 2003; Huang et al. 2011) as well as promoting the production of endothelin‐1 (ET‐1) (Kähler et al. 2001). Importantly, endothelial cells are exquisitely sensitive to flow, and severe reductions in shear stress at the capillary level provoke a multitude of changes within the intracellular milieu that include the overproduction of ROS and cell death (Wei et al. 1999; Paravicini and Touyz 2006; Nayak et al. 2011). Thus, GC‐mediated superoxide production is a plausible mechanism by which elevated GCs could lower blood flow, induce capillary rarefaction and contribute to hypertension.

The microvascular perfusion heterogeneity that accompanies excessive vasoconstriction and capillary rarefaction also increases tissue oxidative stress (Muller et al. 2012; Butcher et al. 2013), thereby participating in a positive feedback loop through which oxidative‐stress damage is amplified. In fact, we previously reported that cotreatment with the *α*‐1 adrenergic receptor antagonist prazosin prevented GC‐induced capillary rarefaction in rodent skeletal muscle (Mandel et al. 2016), demonstrating the importance of microvascular blood flow as a determinant of capillary homeostasis. Interestingly, *α*1 adrenergic receptor activation also has been reported to enhance NADPH oxidase‐dependent superoxide production (Bleeke et al. 2004), suggesting that prazosin could directly influence local superoxide levels in addition to its established role in the inhibition of norepinephrine‐dependent vascular tone.

The main goal of this study was to determine if sustained GC excess enhances superoxide‐dependent oxidative stress within rat skeletal muscle and if this contributes to the altered microvascular content. We hypothesized that tempol, a superoxide dismutase mimetic that lowers superoxide levels, would be sufficient to prevent GC‐mediated capillary rarefaction. Furthermore, we hypothesized that the positive influence of *α*1‐adrenergic receptor antagonism on the maintenance of capillary content includes lowering of skeletal muscle oxidative stress.

## Methods

All animal experiments were approved by York University Animal Care Committee and conducted in accordance with the Canadian Council for Animal Care Guidelines.

### Animal protocols

**Figure 1:**
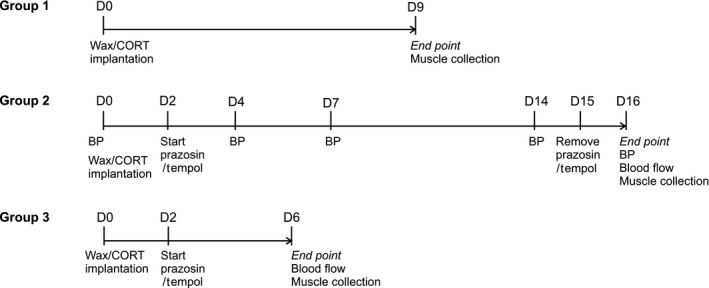
Schematic representation of the experimental time course. The three experimental groups used in this study are depicted to illustrate the time course and assessments conducted. D, day; BP, blood pressure.

Three groups of rats (*N* = 62) were used in this study. For all groups, male Sprague–Dawley rats (initial weight 200–250 g, ~ 6–8 weeks of age) were purchased from Charles River Laboratories (Montreal, QC, Canada). Rats were housed in the York University Vivarium in 12‐h light–dark cycle. All animals were fed a standard rodent chow diet (14% fat, 54% carbohydrate, 32% fat; 3.0 calories/g) *ad libitum*. After 7 days acclimation, animals were anesthetized by isoflurane inhalation and were implanted with four 100 mg wax (control) or CORT pellets (C2505, Sigma‐Aldrich, Oakville, Ontario, Canada) via subcutaneous incision in the mid‐scapular region, as described previously (Shpilberg et al. 2012; Dunford et al. 2017). Rats recovered in individual cages and were given ampicillin (20 mg/kg body weight) in their drinking water for 2 days, after which either regular or medicated water was given. An overview of the experimental time course is shown in Figure 1.

#### Group 1

Referred to as 9D CORT, these rats were from a previously conducted experiment that involved control‐water or CORT‐water treatments for 9 days prior to tissue collection (Mandel et al. 2016; Dunford et al. 2017). Initial mRNA and muscle glutathione assessments (described below) were conducted on *tibialis anterior* (TA) or *gastrocnemius* muscles from this subset of rats (*N* = 10).

#### Group 2

Referred to as 16D, these rats (*n* = 6/group) were given regular water (con‐water and CORT‐water) or water containing 172 mg/L (or, 1 mmol/L) tempol (#176141, Sigma‐Aldrich Canada) (con‐tempol and CORT‐tempol) or containing 50 mg/L prazosin (P7791, Sigma‐Aldrich Canada) (con‐prazosin and CORT‐prazosin) for 15 days, beginning 2 days post pellet implantation. Based on an average daily water consumption of 50 mL, rats consumed approximately 9 mg/day tempol or 2.5 mg/day prazosin. Tempol, a stable cell permeant piperidine nitroxide that acts as a superoxide dismutase mimetic (Wilcox and Pearlman 2008), was used to lower endogenous levels of superoxide radicals. This dose of tempol was reported to significantly lower blood pressure in male Sprague–Dawley rats (Zhang et al. 2004). Prazosin, a selective *α*‐1 adrenergic receptor antagonist, was utilized to prevent GC‐induced capillary rarefaction in rats, as previously reported (Mandel et al. 2016). Systolic blood pressure and heart rate were assessed via noninvasive tail plethysmography (LE 5001 Pressure Meter, Harvard Apparatus). Assessments were made at D0, prior to pellet implantation, as well as on D2, D4, D7, and D14 post pellet implantation. On D15, water containing tempol and prazosin was replaced with untreated drinking water. The final blood pressure assessment (D16) was conducted 18 h after removal of prazosin or tempol from the drinking water. Based on the pharmacokinetics of both tempol and prazosin (Jaillon 1980; Ueda et al. 2003), these drugs would exert minimal functional effects on day 16. In all cases, the animals were lightly sedated (isoflurane inhalation) and body temperature was maintained at the time of assessment. Tissue collection occurred on D16.

#### Group 3

Based on findings from the 16D rats, a final group of rats (referred to as 6D) was utilized to analyze short‐term treatment effects of tempol and prazosin. Control and CORT rats (*N* = 20) were concurrently treated with water, or water containing tempol (172 mg/L) or prazosin (50 mg/L), and assessments of muscle blood flow, glutathione, and mRNA levels were conducted 6 days post pellet implantation.

### Tissue isolation

At the end of each protocol, hind limb skeletal muscles were removed under isoflurane anesthesia. Muscles were weighed and snap frozen in liquid nitrogen for RNA and protein analyses or frozen in liquid nitrogen cooled isopentane for histology.

### Hind limb muscle blood flow

Skeletal muscle blood flow was assessed in 6D and 16D rats, using a microsphere protocol modified from (Deveci and Egginton 1999). Briefly, animals were anesthetized with isoflurane and fluorescent microspheres (500,000 spheres in 0.5 mL PBS) (Molecular Probes, FluoSpheres 15 *μ*m diameter, #F8844) were injected directly into the left ventricle. The brain, *extensor digitorum longus* (EDL) and *soleus* muscles were immediately collected and flash frozen in liquid nitrogen. For analysis, tissue was incubated in 2mol/L 0.5% tween ethanoic KOH for 24 h at 37°C. After digestion, tubes were spun at 3000RPM for 15 min, followed by two ethanoic‐tween washes. The microsphere pellet was resuspended in xylene and the fluorescent signal was detected with a microplate reader (Cytation^™^ 3, Biotek, Vermont USA). The normalized fluorescent intensity (FI) was calculated relative to tissue mass (FI/100 g tissue). In the 16D experiment, relative blood flows were estimated by expressing the normalized FI of EDL and soleus muscles as a ratio to normalized brain FI, to discount the impact of artifacts associated with animal‐to‐animal variability in the microsphere injection. In the 6D experiment, EDL blood flow was expressed as a ratio to that of soleus (which was found not to be altered by 16D CORT treatment).

### RNA extraction and real‐time qPCR

RNA was isolated from TA muscle using the Qiagen RNeasy Fibrous Tissue Mini Kit (74704, Qiagen, Toronto, ON Canada) as per the manufacturer's instructions. RNA was isolated from cultured endothelial cells using cells to cDNA lysis buffer (#AM8723, Invitrogen Canada; Burlington, ON Canada). RNA was reverse transcribed using MMLV reverse transcriptase (New England Biolabs, Whitby ON Canada). cDNA were analyzed by Taqman qPCR using qPCR mastermix (Invitrogen Canada) and Taqman probes for rat HPRT (Rn01527840), eNOS (Rn02132634), *α*1A adrenergic receptor (Rn00567876), NOX1 (Rn00586652_m1), NOX2 (gp91^phox^, Rn00576710_m1), NOX3 (Rn01430441_m1), p47^phox^ (Rn00586945_m1), and ET‐1 (Rn00561129_m1) using the ABI 7500 Fast PCR system (Invitrogen Canada). For each sample, the comparative Ct method was used to determine mRNA expression of target genes relative to the housekeeping gene HPRT and expressed as 2^−ΔCt^.

### Oxidative stress

Protein carbonylation was assessed as an indirect measure of accumulated oxidative stress (Davies et al. 1999) using a Protein Carbonyl Colorimetric Assay Kit (# 10005020; Cayman Chemical, Ann Arbor MI). Briefly, 100 mg of tissue was extracted from the TA or *gastrocnemius* muscle in 1× PBS containing 1 mmol/L EDTA. Protein carbonyl content was assessed as per the manufacturer's instructions and was expressed as nmol/mg of total protein.

### Skeletal muscle glutathione

Skeletal muscle glutathione was assessed in the *gastrocnemius* muscle following homogenization in a 50 mmol/L Tris‐based buffer containing 20 mmol/L boric acid, 2 mmol/L L‐serine, 20 *μ*mol/L acivicin and 5 mmol/L N‐ethylmaleimide. Muscle homogenate was acidified using trichloroacetic acid for reduced glutathione (GSH) determination, and 15% perchloric acid (PCA, Caledon Laboratories Ltd, Georgetown, Canada) for oxidized glutathione (GSSG). The TCA‐acidified muscle sample was then used for GSH analyses by HPLC (Agilent 1100) with column separation performed using a Zorbax high performance analytical 4.6 × 150 mm 5 *μ*m column (Agilent, Santa Clara, USA). PCA‐acidified muscle sample was diluted (1:5) in 0.5mol/L NaOH. Samples were then incubated in the dark with 0.1% O‐pthalymide (OPA, Sigma‐Aldrich, Oakville, Canada) for GSSG analysis. Prior to acid deproteination, a sample of muscle homogenate was removed for protein concentration analysis using a Pierce BCA protein assay kit (Thermo, Fisher Scientific, Waltham, USA). All values were referenced to protein concentration and reported in *μ*mol/g protein. GSH was determined as previously described (Giustarini et al. 2013). Samples were run in a 0.25% glacial acetic acid mobile phase with 6% acetonitrile. GS‐NEM was detected using a modular variable wavelength detector at 265 nm wavelength. Values were referenced to a standard curve of reduced glutathione (Sigma‐Aldrich, Oakville, Canada).

Oxidized glutathione was determined by separation by HPLC as previously described (Kand'ár et al. 2007). A 25 mmol/L Na_2_HPO_4_ in HPLC grade water with 15% methanol was used for mobile phase. Following column separation, samples flowed through a flow‐through cuvette (FireflySci 8830, New York, USA) in a PTI QuantaMaster 40 spectrofluorometer (Horiba, New Jersey, USA). GS‐OPA conjugate was excited at 350 nm and emission was detected at 420 nm.

Total glutathione was calculated as the sum of GSH and 2×GSSG values.

### Muscle histology

A 10 *μ*m cryosections of TA muscle were fixed with 3.7% paraformaldehyde and stained with fluorescein isothiocyanate‐conjugated *Griffonia simplicifolia* isolectin B4 (1:100; Vector Laboratories, Burlington ON, Canada) and anti‐smooth muscle actin‐Cy3 (1:300; C6198, Sigma‐Aldrich, Oakville ON, Canada). Sections were viewed using a Zeiss M200 inverted microscope with 20× objective. Images were captured using a cooled CCD camera using Metamorph imaging software. Capillary‐to‐fiber (C:F) counts were averaged from three to four independent fields of view per rat by a blinded observer.

### Cell culture experiments

Skeletal muscle microvascular endothelial cells were isolated from the TA muscle of male Sprague–Dawley rats (~8 weeks old) as described previously (Han et al. 2003). Cells were cultured with Dulbecco's Modified Eagle Medium (Invitrogen) supplemented with 10% heat denatured FBS, 1 mmol/L sodium pyruvate, 1 mmol/L Glutamax (Invitrogen), 50 units penicillin, 0.5 mg/mL streptomycin, and 1.25 μg/mL Fungizone (Gibco). Cells were used for experiments between passages 4 and 6. Endothelial cells were plated on 12‐well plates. Endothelial cells were treated with 600 nmol/L CORT for 48 h (Control or CORT‐treated), a dose that previously was shown to inhibit angiogenic behavior (Small et al. 2005; Shikatani et al. 2012). RNA was isolated from cultured endothelial cells using cells to cDNA lysis buffer (#AM8723, Invitrogen Canada; Burlington, ON Canada).

### Statistical analysis

Results were presented as mean ± SEM and analyzed by t‐test or using one‐ or two‐way ANOVA with subsequent Bonferroni post hoc tests (Prism4; Graphpad software Inc; La Jolla, CA). Systolic blood pressure was analyzed using a proc mixed of general linear model for a repeated measures analysis using SAS software, to account for repeated measures, the various conditions and treatment groups. *P *<* *0.05 was considered statistically significant.

## Results

### CORT induced oxidative stress within skeletal muscle

**Figure 2:**
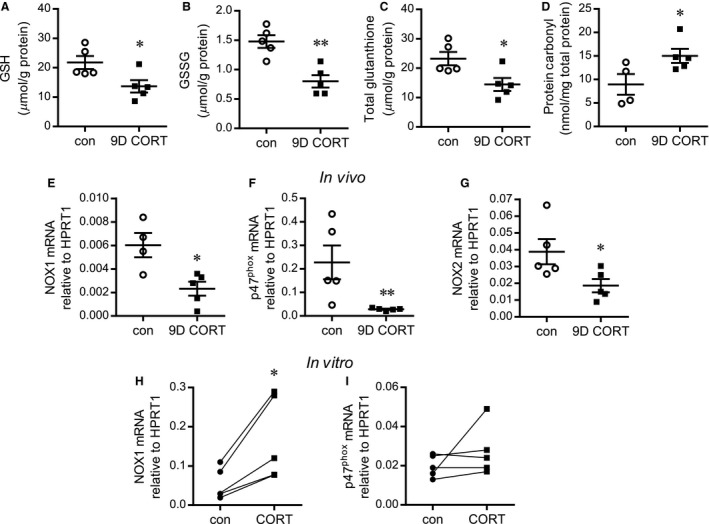
CORT reduces reactive oxygen species buffering and increases oxidative stress within skeletal muscle. Protein was extracted from the gastrocnemius muscle of control (con) or 9D CORT‐treated rats to assess reduced glutathione (GSH) (A), oxidized glutathione (GSSG) (B), total glutathione (C), and protein carbonylation (D). *P <* *0.05 relative to controls (*n* = 5). RNA was isolated from the TA muscles of these rats and analyzed by Taqman qPCR to quantify the mRNA levels of NOX1 (E), NOX‐2 (F) ,and p47^phox^ (G), expressed as 2^−ΔCt^ relative to the housekeeping gene HPRT1. *P <* *0.05 relative to control (*n* = 5). Rat skeletal muscle microvascular endothelial cells were treated for 48 h with CORT (600 nmol/L) followed by assessment of mRNA levels of NOX1 (H) and p47^phox^ (I) using Taqman qPCR (expressed as 2^−ΔCt^, relative to HPRT). *P <* *0.05 relative to control, Students paired t‐test (*n* = 5).

Elevated oxidative stress can result from either an increase in oxidant production or decrease in redox buffering (e.g., glutathione buffering, GSH). The glutathione system is the dominant intracellular buffer of oxidants and is critically involved in protecting cells from ROS‐mediated damage (Dringen 2000). Total glutathione, GSH (reduced glutathione), and GSSG (oxidized glutathione) were significantly lower with 9D CORT treatment (Fig. 2A–C), indicating a substantial diminution in the skeletal muscle capacity for ROS buffering. Contemporary definitions of oxidative stress include consideration of macromolecular damage by oxidants (Jones 2008) such as irreversible protein oxidative modifications. In this regard, irreversible protein carbonylation increased significantly in the muscles of rats following 9D CORT treatment (Fig. 2D).

NADPH oxidase is a major producer of cellular superoxide radicals (Bedard and Krause 2007). A bioinformatics search using the MultiTF function within ECR browser (Ovcharenko et al. 2004) identified putative multi‐species‐conserved glucocorticoid response elements (GRE) within the promoter regions of *Nox1, Nox2*, and *p47* ^*phox*^, suggesting that they might be modified through GC‐mediated transcriptional regulation. However, *Nox1, Nox2*, and *p47* ^*phox*^ mRNA were significantly lower within whole muscle homogenates after 9D CORT (Fig. 2E–G). *Nox3* mRNA was below the level of detection. Conversely, *Nox1* mRNA was significantly increased in cultured microvascular endothelial cells after 48 h of CORT (Fig. 2H). No significant changes in *p47* ^*phox*^ were seen (Fig. 2I), while both *Nox2* and *Nox3* mRNA were undetectable, in the cultured rat endothelial cells.

### CORT favors vasoconstriction and lowers glycolytic muscle blood flow

**Figure 3:**
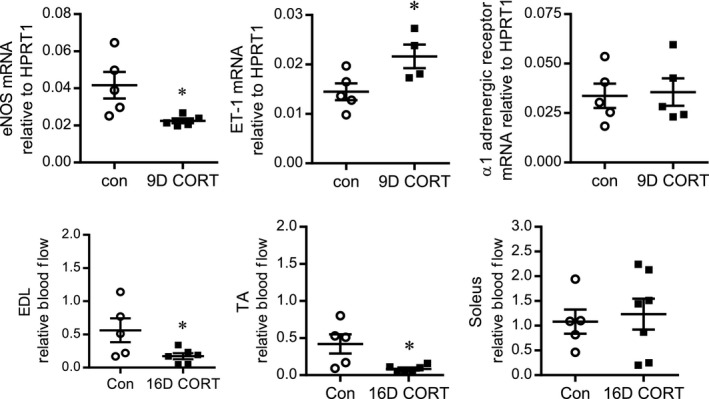
CORT promotes a vasoconstrictor profile within skeletal muscle. RNA was isolated from the TA muscle of control or 9D CORT‐treated rats and quantified by Taqman qPCR to assess the mRNA levels of eNOS (A), ET‐1 (B), and the *α*1Α‐adrenergic receptor (C), expressed as 2^−ΔCt^ relative to the housekeeping gene hypoxanthine‐guanine phosphoribosyltransferase‐1 (HPRT1). *P < 0.05 compared to control (con); Student's unpaired t‐test (*n* = 5). Skeletal muscle blood flow was assessed within the EDL (D), the TA (E), and the soleus (F) using fluorescent microspheres, expressing the signal (AU/mg tissue) relative to the brain. The influence of 16D CORT was analyzed by Student's unpaired t‐test. *P <* *0.05 compared to 16D CORT (*n* = 7–8).

Endothelial nitric oxide synthase (eNOS), endothelin‐1 (ET‐1) and the *α*1 adrenergic receptor were examined in 9D CORT‐treated rats (Group 1) as vaso‐regulatory factors that might be influenced by oxidative stress. CORT treatments significantly lowered eNOS mRNA while concurrently increasing ET‐1 mRNA within the TA muscle (Fig. 3A and B). However, mRNA of the *α*1A adrenergic receptor was not altered by CORT (Fig. 3C). The altered profile of eNOS and ET1 suggested a stronger vasoconstrictor influence may prevail within the skeletal muscle of GC‐treated animals. To follow up this observation, we assessed skeletal muscle blood flow in rats after 16D CORT treatment (Group 2). CORT significantly decreased blood flow to the EDL (~70%) and TA muscles (~80%), while soleus muscle blood flow was unaffected (Fig. 3C–E).

### Influence of tempol or prazosin treatment on CORT‐induced capillary rarefaction and hypertension

**Figure 4:**
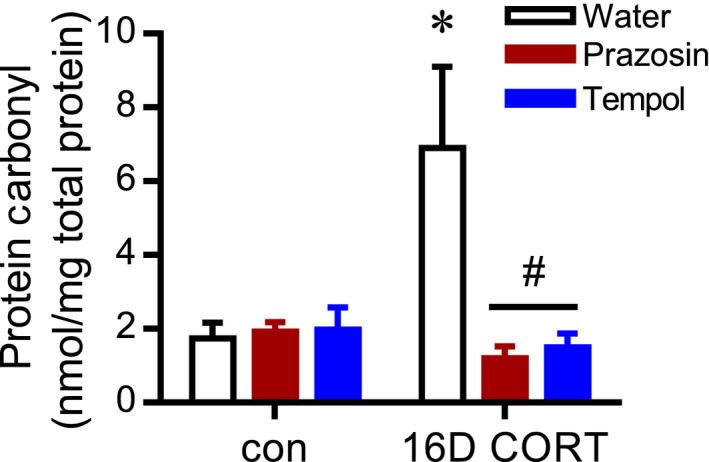
CORT‐induced oxidative stress is ameliorated by tempol or prazosin cotreatment. Protein carbonyl content, an indirect marker of oxidative stress, was assessed in the muscle of 16D CORT‐treated and control (con) animals with or without concurrent prazosin or tempol administration. *P <* *0.05 relative to control group, ^#^ *P *<* *0.05 relative to CORT‐water.

To examine the causal role of CORT‐induced oxidative stress on capillary rarefaction and its plausible effect on hypertension, rats were cotreated with the superoxide scavenger tempol. In addition, some rats were treated with the *α*1‐adrenergic receptor antagonist prazosin, to assess if its previously established capacity to preserve capillary content in the skeletal muscle of CORT‐treated rats is associated with lowering of oxidative stress. As expected, tempol was effective in lowering CORT‐induced oxidative stress, as evidenced by the significant decrease in protein carbonylation with 16D tempol cotreatment when compared to CORT alone (Fig. 4). Notably, prazosin cotreatment also abolished the CORT‐induced increase in protein carbonylation (Fig. 4).

**Figure 5:**
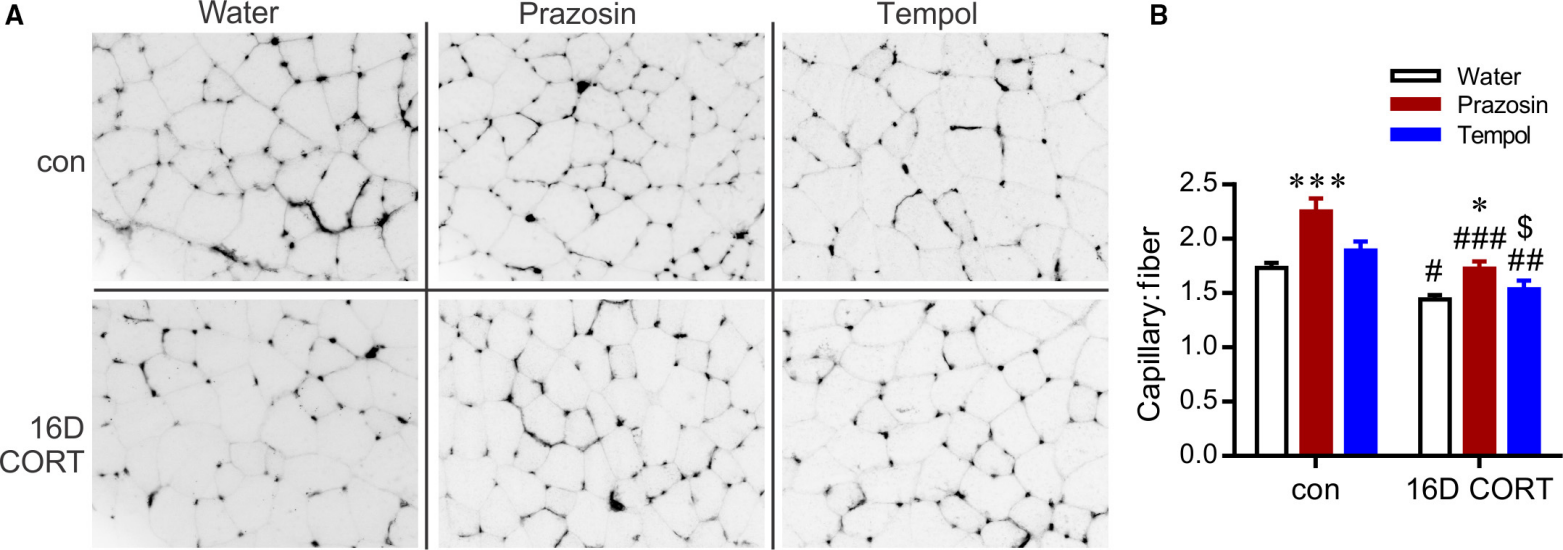
Microvascular rarefaction induced by sustained elevations in CORT is not reversed by tempol treatment. (A) Representative images 16D EDL muscle stained for visualization of capillaries using *Griffonia Simplicifolia* isolectin‐FITC. Inverted gray scale images are displayed to enhance visualization of individual muscle fibers. (B) Capillary‐to‐fiber ratio from 3 to 4 nonoverlapping fields of view was assessed by two‐way ANOVA and subsequent post hoc test. *^,^***P <* *0.05 or 0.001, respectively, compared to respective water group. ^#,^^##,^^###^ *P *<* *0.05, 0.01, and 0.001 compared to respective control group; ^$^ *P *<* *0.01 relative to CORT‐prazosin.

C:F was assessed as a measure of microvascular content in the EDL muscle (Fig. 5A and B). 16D CORT caused a significant reduction in EDL C:F, which was prevented by concurrent prazosin, consistent with previous findings (Mandel et al. 2016). In contrast, tempol treatment did not influence CORT‐induced capillary rarefaction (Fig. 5A and B). Furthermore, prazosin, but not tempol, exerted an angiogenic effect within skeletal muscle of control animals (Fig. 5A and B). The lack of angiogenic influence of tempol was confirmed by subsequent analysis of C:F in the TA muscle (control: 1.71 ± 0.03; control‐tempol:1.73 ± 0.03; CORT: 1.51 ± 0.05; CORT‐tempol: 1.46 ± 0.06).

**Figure 6:**
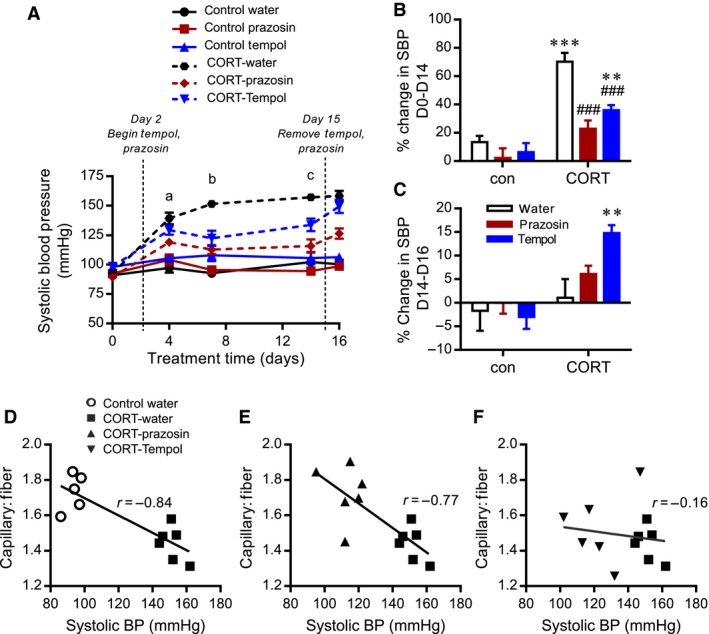
CORT‐induced hypertension is improved by concurrent tempol or prazosin treatment. (A) Blood pressure was measured via tail plethysmography on days 0,4,7,1,4 and 16. A general linear model of repeated measures analysis was used to analyze the blood pressure data over days 0 to 14. D0 systolic blood pressure was not significantly different between the groups (*P *=* *0.11). Systolic blood pressure was not significantly different between the three control groups (con‐water, con‐prazosin, con‐tempol) at any time point, thus for all subsequent analyses, CORT‐groups were compared to a composite of controls. a: D4 blood pressure CORT‐water was significantly different from control groups (*P *<* *0.0001); CORT‐water was significantly different compared to CORT‐prazosin (*P = *0.008) but not CORT‐tempol (*P *=* *0.15). There was no significant difference between CORT‐prazosin and CORT‐tempol (*P *=* *0.08). b: D7 blood pressure was significantly elevated in CORT‐water compared to control animals (*P *<* *0.0001); both CORT‐prazosin and CORT‐tempol were significantly different than CORT‐water (*P < *0.0001 and *P *<* *0.002, respectively) but remained significantly elevated compared to control animals (*P *<* *0.05). c: D14 blood pressure was significantly elevated in CORT‐water compared to control animals (*P *<* *0.0001), and this was significantly improved by both prazosin and tempol treatments (*P *<* *0.0001 and *P = *0.004, respectively). Blood pressure was significantly different between CORT‐prazosin and CORT‐tempol (*P *=* *0.047). B) The percent change in blood pressure (delta systolic blood pressure) between day 0 and day 14 was assessed using two‐way ANOVA and post hoc tests. **^,^ ***P <* *0.01*, P *<* *0.001 compared to corresponding control group, ^###^ *P *<* *0.001 compared to CORT‐water. C) Blood pressure was reassessed on D16, 18 h after removal of prazosin or tempol. The percent change in blood pressure relative to D14 was determined for each treatment group. Data were analyzed by two‐way ANOVA and post hoc tests; **P <* *0.01 versus corresponding control. (D–F) Correlation analysis was conducted utilizing D7 systolic blood pressure and D16 C:F values to compare control‐water and CORT‐water groups (D; *r* = −0.84; *P *<* *0.05), CORT‐water and CORT‐prazosin groups (E; *r* = −0.77; *P *<* *0.05) and CORT‐water and CORT‐tempol groups (F; *r* = −0.16; *P *>* *0.05).

CORT treatment caused a significant 1.5‐fold elevation in systolic blood pressure as early as D4 posttreatment, and increasing 1.7‐fold after D14, compared to D0 (Fig. 6A). Heart rate was not significantly altered by CORT treatment at any time point (data not shown). The CORT‐induced increase in systolic blood pressure was significantly improved in both CORT‐prazosin and CORT‐tempol‐treated groups (Fig. 6A and B). The prazosin‐induced reduction in systolic blood pressure was detectable by D4 of treatment, whereas tempol treatment did not exert a significant effect until the D7 time point. Remarkably, the CORT‐prazosin‐ treated group maintained a near normal (control‐water) systolic blood pressure, whereas systolic pressure in the tempol‐treated rats remained significantly elevated above control‐water groups at all time points (Fig. 6A and B). Neither tempol nor prazosin significantly influenced systolic blood pressure in control rats (Fig. 6A and B).

The depressor influence of prazosin and tempol on systolic blood pressure may be a consequence of improvements in skeletal muscle vascular resistance either through enhanced vasodilation or due to structural remodeling of the microvasculature. We reasoned that reassessment of blood pressure 1 day after the removal of prazosin and tempol could serve to distinguish between functional and structural improvements since acute vasodilatory signals would depend on the continued presence of the drugs, while structural remodeling should continue to exert a positive influence on systolic blood pressure in the absence of the drug. At day 16, 1 day after washout of prazosin and tempol, systolic blood pressure was consistently elevated in the CORT‐water group, as expected given that CORT remained present. Systolic blood pressure also was unaltered in all control groups (Fig. 6A, C). Systolic blood pressure in CORT‐prazosin animals was not different at day 16 compared to day 14, and remained significantly lower than in the CORT‐water group (Fig. 6A, C). However, the removal of tempol resulted in a significant rise in systolic blood pressure compared to all other groups, such that it was no longer different from that of CORT‐water‐treated animals (Fig. 6A, C). This suggested that the beneficial effect of tempol on systolic blood pressure was transitory, rapidly reversible, and likely nonstructural.

Considering that elevated systolic pressure has been identified as a factor that causes capillary rarefaction, we correlated D7 blood pressure (a time point prior to evident capillary rarefaction) with D16 C:F (as shown in Fig. 5). A significant negative correlation between systolic blood pressure and EDL C:F was detected when comparing control with CORT (*r* = −0.84; Fig. 6D) and CORT‐water with CORT‐prazosin (*r* = −0.77; Fig. 6E). However, this relationship was not maintained in the CORT‐tempol group (*r* = −0.16; Fig. 6F), which may suggest that systolic blood pressure *per se* is not the key determinant of muscle capillarization.

### Prazosin, but not tempol, restores ET‐1 mRNA and EDL blood flow in CORT‐treated rats

**Figure 7:**
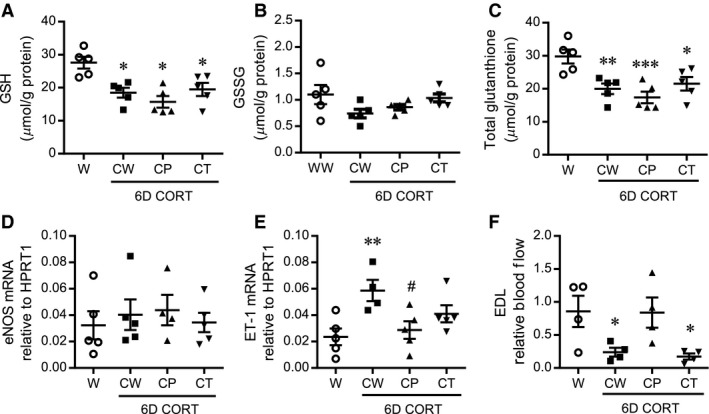
Neither prazosin nor tempol improves glutathione buffering capacity but prazosin restores ET‐1 mRNA and muscle blood flow in CORT‐treated rats. Rats were treated with CORT for 6 days (CW) or were treated concurrently with prazosin (CP) or tempol (CT), and compared to a control‐water (W) group. Reduced glutathione (GSH) (A), oxidized glutathione (GGSG) (B), and total glutathione (C) levels were assessed in gastrocnemius muscles *^,^**^,^ *** *P *<* *0.05, *P *<* *0.01, *P *<* *0.001 relative to control (W), respectively; one‐way ANOVA (*n* = 6). RNA was isolated from the TA muscle, and eNOS (D) and ET‐1 (E) mRNA were quantified by Taqman qPCR, expressed as 2^−ΔCt^ relative to HPRT1. *,# *P < *0.05 compared to control‐water (W) and CORT‐water (CW) groups, respectively; one‐way ANOVA (*n* = 4–5). Skeletal muscle blood flow was assessed using fluorescent microspheres (E). Microsphere deposition in the EDL muscle was normalized to that of the soleus muscle within each animal. The influence of CORT was analyzed via one‐way ANOVA. *P <* *0.05 compared to control‐water (W); *n* = 4.

To further examine differences between prazosin and tempol treatment that could underlie their differential influence on CORT‐induced microvascular rarefaction, we assessed a separate group of rats treated with CORT for 6 days (6D), reflecting a time point at which systolic blood pressure was elevated but that preceded detection of capillary rarefaction. Neither prazosin nor tempol treatment reversed the CORT‐induced decline in GSH, GSSG, or total glutathione content (Fig. 7A–C) in 6D CORT‐treated rats. These findings indicate that the effects of tempol and prazosin are not due to enhanced oxidant buffering. Additionally, eNOS and ET‐1 mRNA levels were assessed in the muscles from these animals. While no significant changes were noted in eNOS mRNA (Fig. 7D), ET‐1 mRNA was significantly elevated with CORT treatment and this was abrogated with prazosin but not tempol cotreatment (Fig. 7E). Blood flow to the EDL was significantly lowered in CORT‐treated rats and this effect was prevented in rats receiving prazosin, but not tempol (Fig. 7F).

## Discussion

This study did not provide support for the hypothesis that oxidative stress promotes skeletal muscle capillary rarefaction of GC‐treated rats. CORT did lower the skeletal muscle oxidant buffering capacity and increased protein carbonylation, an indicator of oxidative damage. Endothelin‐1 mRNA was enhanced and blood flow was lower within the glycolytic muscle in CORT‐treated rats. While the superoxide dismutase mimetic tempol alleviated protein carbonylation, it was ineffective in restoring skeletal muscle blood flow and preventing capillary rarefaction. Conversely, the *α*‐1 adrenergic receptor antagonist prazosin prevented the accumulation of protein carbonylation, improved skeletal muscle blood flow, and prevented capillary rarefaction in GC‐treated rats.

On the basis of prior research linking oxidative stress to capillary rarefaction in various pathophysiological scenarios, we originally postulated that oxidative stress contributed significantly to the capillary rarefaction that occurs within CORT‐treated glycolytic skeletal muscles. Elevated oxidative stress may originate from reduced buffering capacity or increased production of oxidants. Notably, CORT substantially lowered oxidant buffering capacity through depletion of total muscle glutathione, which would increase vulnerability of the muscle to the influence of oxidants. Accordingly, we detected an elevation in oxidative stress (in the form of protein carbonylation) within glycolytic skeletal muscle after 9D and 16D of CORT treatment. Although CORT treatment decreased the transcript levels of NADPH oxidase subunits, *Nox1*,* Nox2*, and *p47* ^*phox*^ in whole muscle lysates, the increase in endothelial cell *Nox1* mRNA in cultured cells suggests that CORT may also increase vascular superoxide radical production. These data suggest that the GC influences on NOX expression may differ between cell types and may localize to the vascular compartment. Similar to our findings, GC treatment was reported to increase NOX2 protein levels in cultured endothelial cells (Muzaffar et al. 2005) and to promote oxidative stress and lower the endothelial‐dependent vasodilatory capacity of arterioles (Schäfer et al. 2005).

The concurrent decrease in eNOS and increase in ET‐1 mRNA detected in 9D suggests that excess CORT promotes a vasoconstrictor influence within the skeletal muscle microcirculation. This was further evidenced by a lower blood flow to TA and EDL muscles. GC‐mediated oxidative stress has been reported to decrease NO levels (Iuchi et al. 2003) and to increase production of the vasoconstrictor ET‐1 (Kähler et al. 2000), consistent with our observations. Thus, it is plausible that the increased oxidative stress detected in this study contributes to alterations in the vasoconstrictor‐vasodilator balance.

Tempol effectively alleviated protein carbonylation in the skeletal muscle of CORT‐treated rats, suggesting superoxide was causal of carbonylation based on its action as a superoxide dismutase mimetic. It is recognized that the dismutase activity of tempol results in the production of hydrogen peroxide (Wilcox and Pearlman 2008). This suggests that the rescue of protein carbonylation by tempol is related to modulation of the levels of superoxide rather than hydrogen peroxide. It should be noted that the exact source of superoxide manipulated by tempol was not determined in this study but may include xanthine oxidase or NADPH oxidase. Mitochondria are also a source of superoxide but it is unlikely that this source was targeted given that tempol is not targeted to the mitochondria, and it is thought that superoxide does not cross the mitochondrial membrane (Jones 2008). Tempol also has been shown to react directly with nitric oxide‐derived oxidants (Augusto et al. 2008), which would influence local levels of nitric oxide and peroxynitrite. Thus, it remains to be determined whether the effects of tempol were due solely to its dismutase activity or if it also reflected alterations in nitric oxide‐derived oxidants.

Interestingly, prazosin also prevented CORT‐induced protein carbonylation. Neither tempol nor prazosin reversed the depletion of total muscle glutathione that accompanied CORT treatment, indicating that their protection against protein carbonylation was unrelated to altered regulation of glutathione buffering itself. While the *α*1‐adrenergic receptor antagonist is not conventionally viewed as having anti‐oxidant action, there is a substantive body of evidence that *α*‐adrenergic receptor activation promotes oxidative stress in vascular smooth muscle and cardiomyocytes (Xiao et al. 2002; Bleeke et al. 2004; Hao et al. 2006; García‐Cazarín et al. 2008). Considering that restriction of blood flow promotes tissue oxidant stress (Gute et al. 1998; McDonagh and Hokama 2000; Lejay et al. 2014), it also is plausible that the beneficial influence of prazosin on protein carbonylation occurs as an indirect consequence of improving blood flow to the muscle. Nonetheless, we did not find evidence to support the central hypothesis that superoxide‐induced oxidative stress is a direct mediator of skeletal muscle capillary rarefaction in CORT‐treated rats. Despite its efficacy in lowering protein carbonylation, tempol failed to prevent CORT‐induced capillary rarefaction. This observation also indicates that the positive influence of prazosin on capillary content is not dependent on its capacity to modulate ROS‐induced oxidant stress.

Microvascular rarefaction has been positioned as both a cause and an effect of increased arterial pressure (Greene et al. 1989; Hansen‐Smith et al. 1996; Noon et al. 1997; Humar et al. 2009; Paiardi et al., 2009; Estato et al., 2013). Considering that detectable capillary rarefaction substantially lagged behind the rapid increase in systolic blood pressure in this rodent model, our data appear to be consistent with the concept that capillary rarefaction occurs as a consequence of augmented systolic blood pressure. However, this explanation is insufficient to fully explain our data, considering that both tempol and prazosin lowered systolic blood pressure markedly in GC‐treated rats*,* while only prazosin alleviated capillary rarefaction. Although C:F varied inversely with systolic blood pressure in control‐CORT and prazosin‐CORT‐treated rats, there was no relation between C:F and blood pressure in tempol‐CORT‐treated animals. The failure of tempol to prevent capillary rarefaction, despite significantly lowering blood pressure, provides strong indication that systolic pressure is not the sole, or direct, driver of capillary rarefaction in GC‐treated rats.

It is notable that the influence of tempol and prazosin on capillary content in CORT‐treated rats correlated most closely with each drug's efficacy in improving muscle blood flow; tempol did not alleviate the impairment of blood flow to the EDL muscle and the increase in ET‐1 mRNA after 6 days of CORT treatment, a time point prior to the detectable occurrence of capillary rarefaction. Conversely, prazosin improved muscle blood flow and restored ET‐1 mRNA levels in the GC‐treated rats. Similar to our findings, tempol treatment failed to improve skeletal muscle blood flow in rats with metabolic syndrome (Frisbee 2005; Frisbee et al. 2011). The same investigators recently demonstrated that adrenergic tone rather than elevated oxidant stress exerted the greatest impact on hyperemic responses in a rat model of metabolic syndrome, although antioxidant (tempol) treatment did improve muscle performance, potentially through fine‐tuning of flow distribution within the muscle (Lemaster et al. 2016). In sum, these reports and our current data indicate that superoxide scavenging by itself does not induce substantive increases in muscle blood flow. However, it is relevant to note that the dismutase‐dependent production of hydrogen peroxide could alter the overall vascular influence of tempol. Hydrogen peroxide itself is a vasoactive molecule that has been demonstrated to exert vascular bed‐ and concentration‐dependent vascular effects that range from vasodilation to vasoconstriction (Cai 2005; Lucchesi et al. 2005). Thus, it is possible that improvements in skeletal muscle flow may have been detectable with higher (or lower) doses of tempol. Overall, our findings serve to strengthen the concept that the GC‐mediated influence on skeletal muscle vascular tone, rather than oxidative stress *per se*, is an important contributor to CORT‐induced capillary rarefaction.

Blood flow is recognized to be a physiological regulator of capillary homeostasis. Particularly evident during development, flow‐dependent microvascular network remodeling involves the “pruning” (or rarefaction) of capillaries with insufficient flow (Chen et al. 2012; Udan et al. 2013) as well as the luminal expansion or division of capillaries with excessive flow (Styp‐Rekowska et al. 2011). Recent work provided evidence that blood flow is an effective modulator of capillary growth and regression within the skeletal muscle of healthy adult rats, demonstrating that a sustained elevation in blood flow induced capillary growth and that return of blood flow to basal levels provoked capillary loss (Egginton et al. 2016). Extending this observation, our data suggest that the extent of capillary rarefaction that occurs under pathological conditions such as systemic hypertension (Chen et al. 1981; Cheng et al. 2008) is linked directly to the magnitude by which capillary blood flow has been compromised.

The efficacy of prazosin in improving blood flow and preventing capillary rarefaction in the EDL may be attributed to the high density of *α*1‐adrenergic receptors within the skeletal muscle microcirculation (Al‐Khazraji et al. 2015) coupled with the tonic release of norepinephrine from sympathetic nerve endings under resting conditions (Korthuis 2011). While this may suggest that excessive *α*1 adrenergic receptor signaling contributes significantly to the vascular pathology seen in the CORT‐treated rats, we do not think that this is the case. First, we did not detect a change in the mRNA levels of the *α*1A adrenergic receptor in muscle from CORT‐treated animals, although previously researchers have reported an increased transcription of *α*1B adrenergic receptor mRNA with dexamethasone treatment (Sakaue and Hoffman 1991). We cannot exclude that there was an increase in receptor density and/or downstream signaling in these animals, as has been shown for *β* adrenergic signaling in cultured cells treated with dexamethasone (Rodan and Rodan 1986). However, an important point of consideration is that prazosin treatment restored muscle blood flow and prevented capillary rarefaction, but it failed to enhance muscle blood flow above basal levels or to induce angiogenesis in CORT‐treated rats as occurs in control rats. This suggests that signal pathways independent of the *α*1 adrenergic receptor continue to restrict the vasodilatory capacity of the microvasculature.

Interestingly, soleus blood flow was not lowered by CORT treatment. The reason for this is not yet known. We previously reported that reported that GC‐induced muscle atrophy in rats is minimal in soleus muscle and that reductions in cross‐sectional area are substantially greater in type IIbx compared to in type I fibers (Shpilberg et al. 2012; Beaudry et al. 2015; Dunford et al. 2017). In rodents, oxidative muscles have higher resting blood flow, greater capillary number, and higher levels of muscle glutathione compared to their glycolytic counterparts, which may influence their tolerance to CORT treatment. Further investigation into the differential responses to GC excess within oxidative and glycolytic muscles, and to determine if these are associated with maintenance of capillary number in oxidative muscle, is warranted.

One caveat to the approach of this study is that markers of oxidative stress and glutathione levels were analyzed on different muscles than those utilized for capillary and blood flow analyses. Because all assessments were conducted on predominantly glycolytic muscles, it is probable that similar patterns would be observable in each muscle, but the potential for muscle‐specific responses to GC treatment cannot be discounted.

In summary, we have demonstrated that sustained elevations in GC lower glutathione‐buffering capacity and augment skeletal muscle protein carbonylation, a measure of oxidative stress. While the blood pressure‐lowering effect of tempol suggests that superoxide‐induced oxidative stress does contribute to the GC‐mediated increase in systemic blood pressure, the failure of tempol to prevent capillary rarefaction provides strong indication that superoxide excess does not mediate this event. Altogether, these data support the concept that local skeletal muscle blood flow is dominantly responsible for the maintenance of microvascular content and suggest that capillary rarefaction is not related to enhanced oxidative stress in the presence of chronic elevations in circulating GC.

## Conflict of Interest

None.
